# Editorial for the Special Issue of Monitoring Anticoagulants

**DOI:** 10.3390/biomedicines10010155

**Published:** 2022-01-12

**Authors:** Jean Amiral

**Affiliations:** SH-Consulting, 78570 Andresy, France; jean.amiral@scientific-hemostasis.com

## 1. Introduction

This Special Issue focuses on monitoring anticoagulant therapies and presents all the most recent updates introduced for laboratory practice, which benefit anticoagulated patients. This issue contains eight articles reporting today’s assay methods available for measuring anticoagulant drug concentrations in plasma and managing their side effects when present. The published reports firstly concern heparin [1] and its complications [2,3], such as heparin-induced thrombocytopenia (HIT), or direct oral anticoagulants (DOACs) [4], which specifically and directly inhibit thrombin or factor Xa (FXa). Other articles review the various approaches for testing ancient, current, and new anticoagulants [5,6]; alternatively, they discuss the specific issues related to the management of critically ill patients who are anticoagulated for prophylaxis or for curative treatment of thromboembolic diseases [7]. Lastly, this issue presents a novel study concerning an anticoagulant protein, thrombomodulin (TM), which is available in the extra-cellular recombinant form as a drug, Recomodulin. Beyond anticoagulant properties, it can also inhibit the binding of tumor cells’ β1-integrin to fibronectin, and it therefore has the potential to reduce and control spreading of malignant tissues [8]. This Special Issue then documents some aspects of today’s state of the art monitoring of anticoagulant and discusses issues and strategies for optimizing patient therapy and controlling side effects.

Although they are more than 70 years old, anticoagulant treatments remain a major therapeutic strategy in many areas of medicine [9], as most pathologies can be associated with an increased risk of developing thrombotic diseases. This is particularly the case in the presence of metabolic disorders, bone fractures, joint replacement therapy, cardiovascular diseases, infectious diseases (as illustrated by the recent outbreak of COVID-19 and the high incidence of blood clotting complications in severely ill patients), malignancy, and snake bites; this list is not exhaustive. An increased incidence of thromboembolic diseases can occur in many pathophysiological contexts when the coagulation balance is disturbed. The introduction of anticoagulants has revolutionized the management of thromboembolic pathology with curative indications and has greatly contributed to preventively reducing its incidence when antithrombotic drugs are used for prophylaxis.

## 2. Discovery and Introduction of Anticoagulant Drugs

Since the introduction of anticoagulant treatments during the first half of the 20th century, mortality and morbidity from thrombotic diseases have been highly reduced. From an historical perspective, anticoagulant activity was first observed to be induced by leeches, which were used from antiquity. The active principle of hirudin was only identified in 1884 (by John Berry Haycraft), purified in the 1950s, and its sequence was established in 1976 [10]. The widely used anticoagulant, unfractionated heparin (UFH), was discovered by McLean and Howell in 1921 and introduced as a therapeutic agent in 1937; however, its clinical use began only in the 1960s and 1970s, with well-established therapeutic protocols and clinical studies defining the posology for the various applications [11,12]. Vitamin K antagonists were discovered when cattle herds were decimated by fatal hemorrhages. The cause of bleeding was understood from the observation of cows eating spoiled sweet cover in meadows during a rainy period; it contained coumarin (warfarin). This compound was first used as a rat poison, before being introduced as a blood-thinning agent in 1952 [13]. Conversely to heparin and hirudin, which are given to patients through intravenous or parenteral routes, warfarin is an oral anticoagulant. 

**Figure 1:**
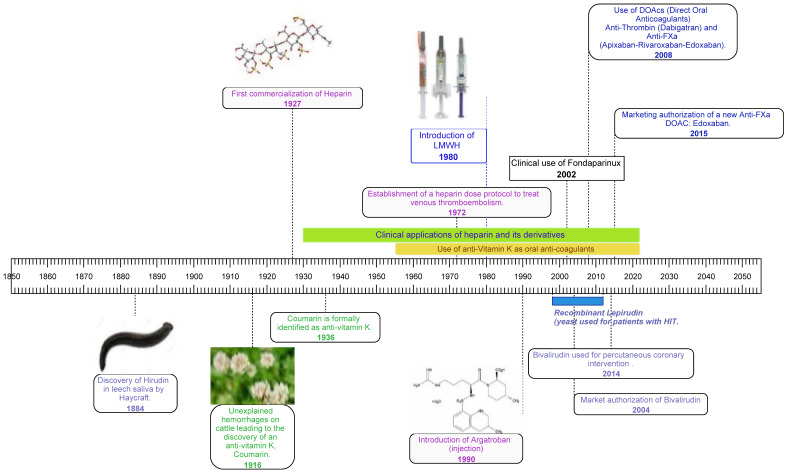
Schema showing the timescale for the discovery and introduction of the various anticoagulants.

Anticoagulant therapy then developed rapidly in the second half of the 20th century, with increasing therapeutic applications. The prognoses of many thrombotic diseases and of thromboembolic complications occurring in various surgeries, especially orthopedic with hip or knee replacement therapies, or bone fracture, were highly improved. Anticoagulants progressively became more sophisticated with the progress of biotechnology and medicine, with a better targeted action mode. Low-molecular-weight heparins (LMWH) and fondaparinux (the pentasaccharide sequence which binds to antithrombin to render it potently anticoagulant) were developed and validated for treating or preventing thrombotic diseases [14]. More recently, direct thrombin and factor Xa Inhibitors were developed and introduced [15]. The first direct thrombin inhibitor (DTI) drugs available were argatroban and hirudin/lepirudin (now withdrawn); these are all injected intravenously and require monitoring of their blood concentration with clotting or chromogenic methods, which measure thrombin inhibition. Later, a truncated molecule derived from hirudin but easier to manage, bivalirudin, was introduced. These past years, many new direct oral anticoagulants have been developed: dabigatran, a DTI; rivaroxaban, apixaban, edoxaban and betrixaban, all direct FXa inhibitors [15]. These drugs are meeting increasing applications for replacing vitamin K antagonists or heparin-like anticoagulants. They now have special long-term indications for the prevention of stroke in patients with atrial fibrillation, and for veinous thrombosis secondary prophylaxis (e.g., idiopathic pulmonary embolism). The history and clinical introduction of anticoagulants is shown on Figure 1.

## 3. Clinical Applications and Monitoring

Anticoagulants are highly efficient for stopping or limiting blood coagulation, and they must be used within a strict, controlled protocol, to avoid bleeding in treated patients. Development of anticoagulant therapy is, therefore, synergistic with that of laboratory tools for monitoring treated patients. The challenge is to obtain potent enough anticoagulation for treating or preventing thromboembolic complications, without exposing patients to excessive bleeding risk. Therapy monitoring has become inseparable from anticoagulant therapy and is often used to adjust posology while preventing severe bleedings [5,6]. Vitamin K Antagonist and heparin therapy are potent anticoagulant therapies, which impact the activity of a wide scope of coagulation proteins. They require a strict adjustment of posology. Furthermore, vitamin K antagonists have a delayed action (2 to 3 days) before reaching the expected anticoagulation level, and diet interferes with their efficacy, as food intake can be a source of vitamin K [5,6]. Laboratory assays are mandatory for reaching therapeutic equilibrium and following drug efficacy and safety over time. Prothrombin time is the assay of choice for drug monitoring and adjustment of posology [5,6]. This assay has been highly standardized since the introduction of an international sensitivity index (ISI) to reduce the reagent-to-reagent variability, and results are given as INR. Unfractionated heparin (UFH) and heparin derivatives, such as low-molecular-weight heparin (LMWH) or fondaparinux, and other polysaccharide anticoagulants, such as Sodium Danaparoid, are widely used for curative or preventive indications [6]. Heparin, especially UFH, has a high anticoagulant potency; however, it has many side effects, such as HIT [2,3], and the associated hemorrhagic risk can be high. Other polysaccharide drugs present a lower bleeding risk and have a more targeted inhibitory impact on some coagulation proteins (especially factor Xa or factor IXa). The half-lives of these drugs (and therefore their blood concentration) can vary according to patients’ conditions, especially in the presence of an impaired renal clearance. 

Heparin-like and polysaccharide anticoagulants are at a constant blood concentration if infused in patients, but they present a peak activity if injected subcutaneously. Blood concentration fluctuates according to the protocol used, drug half-life, and patients’ health status. Blood collection for testing patients must be performed within a well-defined timeframe. Monitoring of these therapies is extremely useful, especially for UFH. Clotting or chromogenic assays are available for evaluating the anticoagulant activity of these polysaccharide anticoagulants [5,6]. The standard approach for drug monitoring tends to rely on anti-factor Xa kinetics assays, and must accurately measure all types of anticoagulants, with a like to like calibrator [1]. Side effects concern mainly HIT, produced by the development of heparin dependent platelet factor 4 (PF4) antibodies. New flow cytometry tools allow detection of the pathogenic effect of these antibodies [2,3] for identification of patients with this complication, which requires an immediate heparin withdrawal and its replacement by another anticoagulant. These tools provide great support to physicians for managing patients, but many questions remain open, as illustrated by Cauchie and Piagnerelli [7].

## 4. Indicators of Anticoagulant Therapy Efficacy

Polysaccharidic anticoagulants and vitamin K antagonists are especially indicated for treating venous thrombosis, whilst anti-aggregant drugs, not treated in this issue, are prescribed to prevent recurrence of arterial thrombosis; the latter is frequently associated with heparins and/or thrombolytics, especially during the acute phase of the disease. They prevent clot growth and allow its dissolution. Laboratory methods are helpful for monitoring the drug’s anti-thrombotic efficacy, or the fibrinolytic activity of thrombolytics. In addition, to adjust posology and to prevent bleeding, practitioners need to know if the anticoagulant treatment, when safely applied to patients, has a healing effect on the disease. This approach is more complex, and no definite tools are yet validated for this follow-up. Blood activation markers are available for detecting some evolving thrombotic states in the clinically silent period, or to identify or exclude thromboembolic diseases in the early stages of their development [16]. Until now, only D-Dimer has met well documented clinical applications for ruling out deep vein thrombosis or pulmonary embolism, or for establishing the duration of an anticoagulant treatment after a thrombotic event. Other markers are available: they objectivate blood activation, platelet or endothelial cell activity or damage, or they are triggers of disease (as is the case for some auto- or allo-antibodies). Laboratory methods allow their measurement for routine applications in a few cases, or for clinical research and epidemiological studies in others. Although their development is restricted, they have promising potential as surrogate markers of disease evolution and therapy efficacy, and they are expected to become more popular if technology allows the performance of easier and automated measurements. 

Laboratory monitoring needs to detect patients with therapy-induced complications early, not only for severe bleeding but also for some side effects such as heparin-induced thrombocytopenia [2,3]. This complication requires an immediate cessation of heparin, and another anticoagulant must be used. The assays which can identify and confirm this complication are discussed in a specific chapter.

## 5. New Directions for Anticoagulation

Anticoagulation has undergone major progresses from the 1950s to the 1980s, especially with the extent of vitamin K antagonists and heparin or polysaccharide-like anticoagulant indications. Many thromboembolic diseases or surgeries, which were known for poor prognoses, are safely managed nowadays. Laboratory tools have allowed establishment of the right protocols and the running of clinical studies for defining the safest and most efficient therapeutic strategies. A new wave of progresses started in the 1990s and 2000s, with the discovery and introduction of clotting enzyme-targeted anticoagulant drugs. They are expected to offer increased antithrombotic activity with fewer side effects, less drug or food interference, and greater ease of administration through oral intake [4]. These anticoagulants do not need any specific monitoring in normal conditions: offer a wide therapeutic window; have predictable pharmacokinetics; present little or no food or drug interferences; and do not need adjustment for age or weight (except in extreme conditions). However, in real life, measuring their drug concentration or activity is required, and this is discussed in this issue [4,5,6]. More particularly, anticoagulants can be prescribed in patients with multiple clinical conditions and therapies, and this renders their surveillance more critical. As an example, anticoagulant therapy is increasingly used in patients with cancer, especially long-term treatments with LMWH, and there are emerging new indications for direct oral anticoagulants (DOACs). The right balance between thrombotic and bleeding risk, needs to be individually assessed. Laboratory investigation of drug concentration and kinetics, and of biomarkers of disease evolution, provide invaluable support for reaching this balance [16].

## 6. Conclusions

This Special Issue has the exhaustive ambition to present and discuss how to safely manage the various anticoagulants that have been, or are being, introduced for treating thromboembolic diseases, thanks to the support of laboratory monitoring, and specific concerns in critically ill patients [7]. The various measurement methods applied to the different anticoagulant drugs are described. Laboratory automation, and how this testing can help to adjust or control therapy, are also discussed. Lastly, the pharmaceutical industry continues to strive for the discovery and validation of new anticoagulants. For the first time, antithrombotic and anticoagulant activities can be partly dissociated in drugs. In addition to DOACs, new antithrombotic drug candidates, targeted to factors XIa or XIIa, are expected to offer efficient antithrombotic activity without exposing patients to bleeding risk. In this early drug development phase, laboratory methods are essential to establish drug efficacy and posology, and to offer surrogate endpoints for clinical trials [16]. Another group of anticoagulant molecules, used as enhanced natural antithrombotic agents, concerns some engineered blood extraction or recombinant proteins, such as antithrombin, activated protein C, thrombomodulin or tissue factor pathway inhibitor. In this issue, the clinical potential for a recombinant thrombomodulin variant, or its truncated form, is discussed [7].

The various articles presented are a review of the specific assays, which are designed for measuring the various anticoagulant drug concentrations in patients’ plasma and monitoring their kinetics. In conclusion, this Special Issue covers various areas of monitoring anticoagulant therapies, from the history of their development to their medical introduction to laboratory testing tools. This issue ends with the new drugs under current discovery or development. We trust that it can offer to the reader a complete scope of available tools for controlling anticoagulant therapy in the 2020s.

## Data Availability

Not applicable.
